# P-1111. Efficacy of Intra-Articular Administration of Rifaquizinone in Rodent Models of Implant Infection Associated with *Staphylococcus aureus*

**DOI:** 10.1093/ofid/ofae631.1299

**Published:** 2025-01-29

**Authors:** William J Weiss, Mark E Pulse, Phung Nguyen, David Valtierra, Kelly Peterson, Adaeze Ogbonna, Lane Beeman, Tianyu Dai, Liaobin Chen, Huan Wang, Zhenkun Ma

**Affiliations:** University of North Texas Health Science Center, Fort Worth, Texas; University of North Texas Health Science Center, Fort Worth, Texas; University of North Texas Health Science Center, Fort Worth, Texas; HSC College of Pharmacy, University of North Texas, Fort Worth, Texas; HSC College of Pharmacy, University of North Texas, Fort Worth, Texas; HSC College of Pharmacy, University of North Texas, Fort Worth, Texas; HSC College of Pharmacy, University of North Texas, Fort Worth, Texas; Zhongnan Hospital of Wuhan University, Wuhan, Hubei, China; Zhongnan Hospital of Wuhan University, Wuhan, Hubei, China; TenNor Therapeutics (Suzhou) Ltd, Suzhou, Jiangsu, China (People's Republic); TenNor Therapeutics, Suzhou Industrial Park, Jiangsu, China (People's Republic)

## Abstract

**Background:**

Rifaquizinone (RFQ, TNP-2092) is a novel multitargeting drug conjugate in development for the treatment of serious or life-threatening bacterial infections including those caused by gram-positive pathogens that have developed or acquired resistance to commonly used antibiotics and those associated with medical devices. RFQ exerts its antibacterial activity by inhibiting RNA polymerase, DNA gyrase, and topoisomerase IV. The current studies were conducted to evaluate the therapeutic efficacy of RFQ following intra-articular (IA) administration in two rodent prosthetic joint infections models with *S. aureus*.

**Methods:**

Female C57BL/6 mice and male Wistar rats were used. Following surgical insertion of a metal wire into the femoral canal, the surgical site was inoculated with approximately 7 log_10_ CFU of an *S. aureus* strain. RFQ and control drugs were IA administered into mice and rats starting 7 days post-surgery once daily for 7 and 14 days, respectively. At 24 hours after the last dose, the wire and femur were aseptically removed and processed for bacterial titers.

**Results:**

In the mice, mean wire and femur counts for the untreated controls at the end of the study were 4.77 and 6.88 log_10_ CFU, respectively. IA delivered RFQ resulted in mean wire and femur counts that were 2.50-2.72 log_10_ CFU and 1.71-3.42 log_10_ CFU lower than the untreated controls, respectively (p ≤ 0.0001). In the rats, mean wire and femur counts for the untreated controls at the end of the study were 6.02 and 5.06 log_10_ CFU, respectively. RFQ resulted in mean wire and femur counts that were 3.32-5.02 log_10_ CFU and 1.21-1.96 log_10_ CFU lower than the untreated controls, respectively (p ≤ 0.01). RFQ administration in the rats resulted in reduced knee swelling and expression of systemic inflammatory markers, a decrease in signs of femoral osteomyelitis and decreased biofilm formation. Doses of RFQ in both species were statistically more effective than vancomycin in reducing the bacterial burden.

**Conclusion:**

The results of these studies indicate that IA injection of RFQ is effective in treating infections related to orthopedic implants. The studies serve as a basis for the use of RFQ in orthopedic practice and the treatment of serious implant-associated infections.

**Disclosures:**

**William J Weiss, MS**, TenNor Therapeutics (Suzhou) Ltd: Investigator **Mark E Pulse, MS**, TenNor Therapeutics (Suzhou) Ltd: Investigator **Phung Nguyen, BS**, TenNor Therapeutics (Suzhou) Ltd: Investigator **David Valtierra, Master**, TenNor Therapeutics (Suzhou) Ltd: Investigator **Kelly Peterson, PhD**, TenNor Therapeutics (Suzhou) Ltd: Investigator **Adaeze Ogbonna, Bachelor**, TenNor Therapeutics (Suzhou) Ltd: Investigator **Lane Beeman, Bachelor**, TenNor Therapeutics (Suzhou) Ltd: Investigator **Tianyu Dai, PhD**, TenNor Therapeutics (Suzhou) Ltd: Investigator **Liaobin Chen, PhD**, TenNor Therapeutics (Suzhou) Ltd: Investigator **Huan Wang, PhD**, TenNor Therapeutics (Suzhou) Ltd: Employee **Zhenkun Ma, PhD**, TenNor Therapeutics (Suzhou) Ltd: Employee

